# Flotation techniques (FLOTAC and mini-FLOTAC) for detecting gastrointestinal parasites in howler monkeys

**DOI:** 10.1186/s13071-017-2532-7

**Published:** 2017-11-23

**Authors:** Mayra Alejandra Alvarado-Villalobos, Giuseppe Cringoli, Maria Paola Maurelli, Aurelie Cambou, Laura Rinaldi, Arturo Barbachano-Guerrero, Roger Guevara, Colin A. Chapman, Juan Carlos Serio-Silva

**Affiliations:** 1 Red de Biología y Conservación de Vertebrados, Instituto de Ecología A.C, Xalapa, 91070 Veracruz, Mexico; 2 Unit of Parasitology and Parasitic Diseases, Department of Veterinary Medicine and Animal Productions, University of Naples Federico II, Naples, Italy; 3 ENSAIA (Ecole National e Supérieure d’Ágronomie et des Industries Alimentaires), Vandoeuvre-lés-Nancy, France; 4 Laboratorio de Medicina de Conservación, Departamento de Estudios de Posgrado e Investigación, Escuela Superior de Medicina, Instituto Politécnico Nacional, México, Mexico; 5 Red de Biología Evolutiva, Instituto de Ecología A.C, Xalapa, 91070 Veracruz, Mexico; 6 Department of Anthropology & McGill School of Environment, McGill University, Quebec, Montreal H3A 2T7 Canada

**Keywords:** Quantitative flotation, Gastrointestinal parasites, Nematodes, Trematodes, Howler monkeys, *Alouatta*

## Abstract

**Background:**

Analyses of environmental correlates of the composition of gastrointestinal parasite communities in black howler monkeys (*Alouatta pigra*) have been hindered by inadequate calibration techniques of detection and quantification methods of the parasites. Here we calibrate samples and compare the likelihood of parasite detection using two flotation techniques, FLOTAC and Mini-FLOTAC, and compare flotation solution, preservation method and dilution ratio for egg detection and counts of the most common parasites (*Controrchis* spp. and *Trypanoxyuris* spp.) in howler monkeys.

**Results:**

For samples preserved in 5% formalin, the Mini-FLOTAC technique was the best option for qualitative and quantitative copro-microscopic analysis. This technique displays an 83.3% and 100% detection of *Controrchis* spp. and *Trypanoxyuris* spp. infections, respectively. For the trematode *Controrchis* spp., more eggs per gram of feces (EPG) were recorded with the flotation solution (FS) #7 (zinc sulfate; specific gravity SG = 1.35) at 1:20 and 1:25 dilution than other methods. By contrast, for the nematode *Trypanoxyuris* spp., the best results were recorded with FS1 (sucrose and formaldehyde; SG = 1.20) at 1:10 dilution.

**Conclusions:**

We recommend the Mini-FLOTAC technique for general use with parasite analysis on frugivore/folivores like the howler monkey, especially if many samples are analyzed. The technique has a high detection rate and the best EPG counts, allowing the qualitative and quantitative analysis of parasite load among the species or populations without the need for specialized equipment.

**Electronic supplementary material:**

The online version of this article (10.1186/s13071-017-2532-7) contains supplementary material, which is available to authorized users.

## Background

The accurate detection of the prevalence and intensity of gastrointestinal parasite infections is key to understanding the effect of parasites on the biology, behavior, and the conservation of hosts. Gastrointestinal parasites are most often surveyed in the feces [1] of wild host populations using light microscopy [2–4], which is particularly effective where the feces of host populations can be identified and collected in the wild, as this eliminates the need to capture or handle host individuals.

An increasing number of studies have focused on the gastrointestinal parasites of howler monkeys (*Alouatta* spp.), in which polyparasitism is common, including helminths, protozoans and acanthocephalans [5]. Among these parasites, trematodes (*Controrchis* spp.) and nematodes (*Trypanoxyuris* spp.) are the most common gastrointestinal parasites of howler monkeys [5, 6]. *Controrchis* spp. are common in most studies [5, 7] and may influence the ecology of the host [8]. By contrast, *Trypanoxyuris* spp. appear to be indicators of ecosystem health with low prevalence in human dominated landscapes and high prevalence in more natural landscapes [9]. Most studies report low levels of *Controrchis* spp. and *Trypanoxyuris* spp. parasitism in the black howler monkey (*Alouatta pigra*) [7, 8], possibly due to unsuitable sampling or analytical methods. Consequently, standardization of the copro-microscopic techniques is essential for the diagnosis of gastrointestinal parasites.

In wild primates, including howler monkeys, parasitic infections are typically detected by identifying eggs, larvae, oocysts, or cysts in the feces of the host by flotation procedures [10–15] or sedimentation techniques [8, 10, 12]. Sodium nitrate (NaNO_3_) is a common flotation solution (FS) for fecal samples from wild primates [16, 17]. However, studies of black howler monkeys (*Alouatta pigra*) have also used sodium chloride (NaCl) [11, 18], zinc sulfate (ZnSO_4_) [13–15], and sucrose (C_12_H_22_O_11_) for samples preserved in 10% formalin [8, 12, 14, 15]. In folivore-frugivore primates, such as *Alouatta pigra* [19–21], identification of parasites in fecal samples is often complicated by the high fiber content of their diet [19], as well as the common presence of pollen, plant tissue, flowers, and invertebrate fragments (accidentally ingested with the plants) [19, 21, 22], all of which can be misclassified as parasitic structures.

We suggest that the development and calibration of parasite copro-microscopic techniques and the standardization of existing methods have not received sufficient attention, particularly as calibration is the foundation of a good diagnosis. The accuracy of fecal egg count (FEC) techniques depends on the analytic sensitivity of the technique, the choice of the flotation solution, and dilution as well as fecal preservation method [1, 4, 23].

The aim of this study is to evaluate the efficiency of detection and counts of the eggs of the most common parasites (*Controrchis* spp. and *Trypanoxyuris* spp.) of howler monkeys, using two novel techniques, FLOTAC and Mini-FLOTAC, six flotation solutions, three different dilutions, and two preservation methods. FLOTAC and Mini-FLOTAC are innovative multivalent quantitative diagnostic techniques sufficiently accurate to estimate the number of parasites in fecal samples [1, 23–25] of 1 g (volume = 10 ml; analytical sensitivity = 1 egg, larvae, oocyst, cyst per gram of feces, EPG/LPG/OPG/CPG) with FLOTAC [1, 23] and up to 0.2 g (volume = 2 ml; analytical sensitivity = 5 EPG/LPG/OPG/CPG) with Mini-FLOTAC.

## Methods

### Sampling methods

**Fig. 1:**
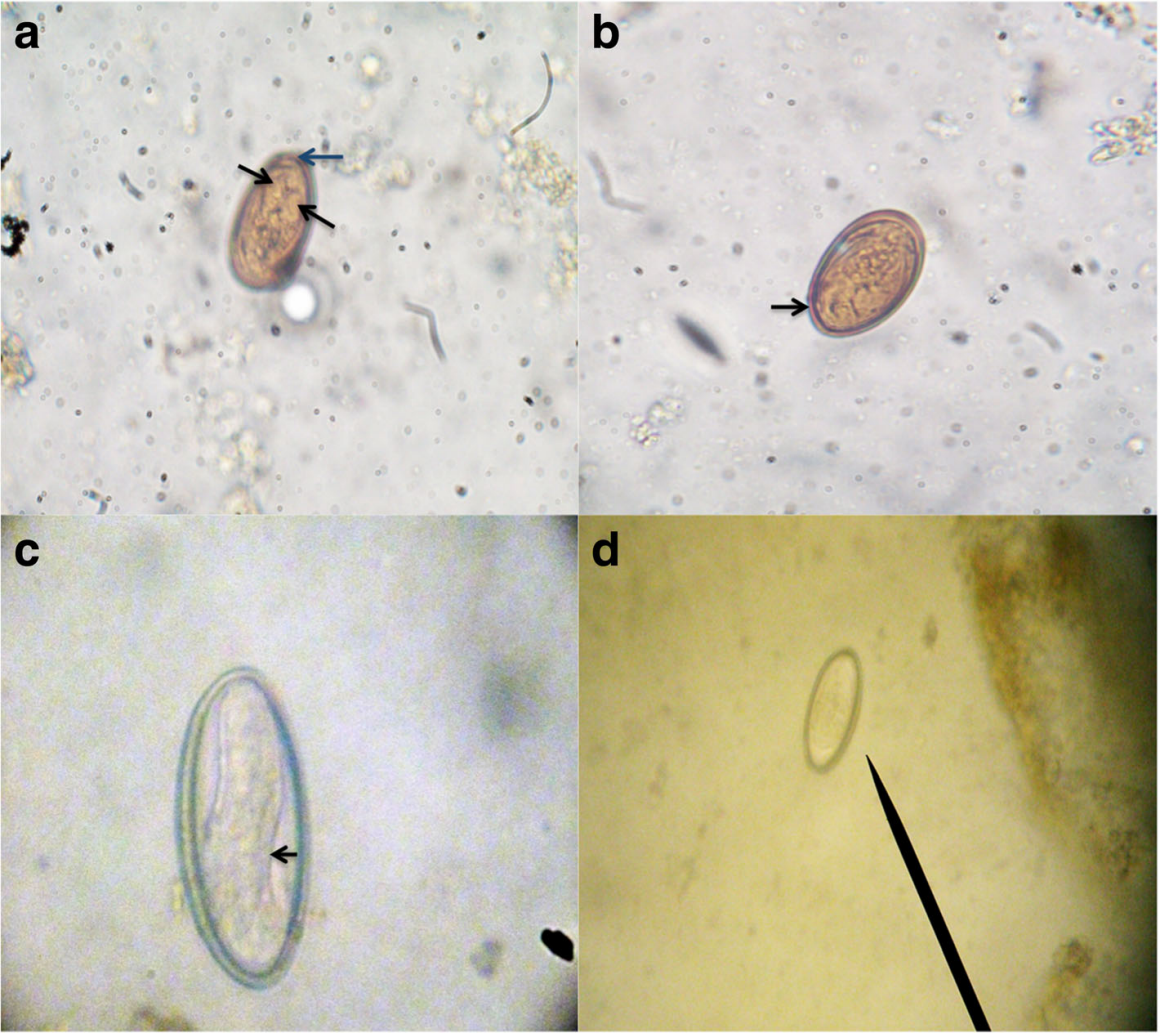
Eggs of *Controrchis* and *Trypanoxyuris.* **a** *Controrchis* spp. egg (400×) measuring 35 × 22 μm. The black arrows indicate the position of the miracidium, the blue arrow shows the operculum. **b** *Controrchis* spp. egg (400×) measuring 35 × 22 μm. The black arrow indicates the thick wall. **c** *Trypanoxyuris* spp. egg (1000×) measuring 36 × 22 μm. The black arrow indicates the larva inside. **d** *Trypanoxyuris* spp. egg (100×) measuring 36 × 22 μm

An additional file shows the “Guide to the recommended quali-quantitative flotation method” which describes the procedure for performing the recommended Mini-FLOTAC basic and dual technique (Additional file 1). A composite from 13 individual fecal samples of wild *A. pigra* individuals (360 g) was performed and divided into sub-samples depending on calibration schemes (Additional file 2: Table S1). Individuals were naturally infected with the trematode *Controrchis* spp. (Fig. 1a) and nematode *Trypanoxyuris* spp. (Fig. 1b). Two fecal preservation methods were used: (i) anaerobic storage by vacuum packing samples (VPF) in the fridge at 4 °C; and (ii) a 5% formalin solution.The VPF samples were analyzed 10 days after collection.

As is typical for howler monkeys, the fecal samples had a high fiber content that made the identification of parasite difficult. Therefore, three different dilution ratios: 1:10, 1:20 and 1:25 (g of feces/ml of water or water plus fixative depending on the calibration scheme) were used to calibrate the samples. The following six FSs were used [1]: FS1 (sucrose and formaldehyde, SG = 1.2); FS2 (sodium chloride, SG = 1.2); FS3 (zinc sulphate, SG = 1.2); FS4 (sodium nitrate, SG = 1.2); FS6 (magnesium sulphate, SG = 1.28); and FS7 (zinc sulphate, SG = 1.35). The FS5 established in the protocol of Cringoli et al. [1] was not implemented because the reagent is corrosive and expensive for routine use. All FSs were prepared at room temperature, and their SG was checked with a hydrometer. For each FS, 6 replicates were performed.

### Calibration

The single composite fecal sample was completely homogenized, divided into two sub-samples of 180 g each for analysis by the FLOTAC and Mini-FLOTAC methods. The sub-samples were further subdivided into two samples of 90 g each, which were either preserved in VPF or 5% formalin at dilution ratio 1:4 (one part of feces and three of fixative). After storage, each 90 g sample was equally divided into three 30 g samples that were diluted at the following ratios 1:10, 1:20, 1:25 (g of feces/ml of water or water plus 5% formalin). Each dilution was sieved (pore size = 250 μm), the waste was discarded and the remaining sample was thoroughly homogenized.

For the FLOTAC method, 108 aliquots (in tubes) preserved by VPF and 108 aliquots preserved in 5% formalin (36 aliquots per dilution) were used. Each aliquot contained 6 ml of fecal suspension. A total of 216 aliquots were centrifuged for three minutes at 1500× *rpm* (170 RCF), and the supernatant discarded, leaving only a pellet in the tube. Each pellet was randomly assigned to one of the six FSs and re-suspended in 6 ml of each solution in a tube. With a pipette, 5 ml (0.5 g) of the resulting fecal suspension were transferred to each centrifuge chamber (*n* = 216 chambers) for analysis by the FLOTAC method, and the samples were centrifuged at 1000× *rpm* (120 RCF) for five minutes. Flotation in a centrifuge causes the debris to sink to the bottom of the chambers and the parasite elements to float to the top under the two ruled grids [1, 23]. After being centrifuged, each 5 ml sample suspension in a chamber was examined with a light microscope at 100× and 400× magnification [1, 23].

For the Mini-FLOTAC method 216 tubes were analyzed (180 g). Samples preserved by VPF (90 g) did not need to be centrifuged and 5 g of feces sample were weighed for every six replicates and homogenized them in their respective FS and dilution. For samples preserved in 5% formalin, 108 tubes (90 g) were centrifuged at 1500× *rpm* for three minutes. The supernatant was discarded, and each tube was filled with 6 ml of the respective FS and dilution. The Mini-FLOTAC chambers were filled with 1 ml (0.1 g) of the homogenized suspension. Finally, the 216 replicates (*n* = 216 chambers) were examined for parasites using a light microscope 100× and 400× magnifications.

A total of 432 tubes (2 flotation methods × 2 preservation methods × 3 dilutions × 6 FSs × 6 replicates per solution) were examined.

### Statistical analysis

Infections are often described as the number of parasitic elements per gram of feces: eggs (EPG), larvae (LPG), oocysts (OPG), or cysts (CPG) per gram of feces. To obtain these values, the analytic sensitivity of the technique must be known. The analytic sensitivity is the ability to detect the smallest number of parasitic elements assessed by a technique, small values mean that the technique has a high analytic sensitivity and is capable of detecting parasitic infections even though the excreted parasites eggs are low. This value is used as a multiplication factor used to express the results in gram of feces. Both techniques used in this paper present a high analytical sensitivity compared to the most used parasitological techniques [1, 25].

For our work using FLOTAC, the multiplication factor is 2 when the dilution ratio is 1:10, 4 using a 1:20 dilution ratio and 5 for 1:25 dilution ratio to obtain our results in EPG. For the Mini-FLOTAC technique the multiplication factors are 10 using 1:10, 20 with 1:20 dilution, and 25 when a 1:25 dilution ratio was used.

The efficiency of the methods was estimated from six replicates (*n* = 6 chambers) as the number of EPG in the flotation solution. The mean EPG, standard deviation (SD) and the coefficient of variation expressed as a percentage [CV (%) = (standard deviation/mean of EPG) × 100] were estimated for each combination of flotation method, fecal preservation method, dilution and FS (Additional file 3: Table S2, Additional file 4: Table S3).

A generalized linear logistic model (binomial error distribution) was used to compare the likelihood of parasite detection (prevalence) among the combinations of flotation methods, fecal preservation methods, dilutions and FSs. Differences in the number of EPG as intensity value (or the capacity of the methods to float parasite elements) were analyzed with generalized linear models using a Poisson distribution and adjusting interactions at fourth level [glm (EPG ~ (method + fecal preservation method + dilution + FS) ^ 4, Poisson)]. Models were checked for homoscedasticity and normality of the residuals. The presence/absence of the parasite and the number of EPG counted (quantitative technique) were the respective response variables. The flotation methods, fecal preservation methods, dilutions, and FSs were explanatory variables. Initially fitted the saturated model and then followed a model simplification procedure to eliminate the explanatory variables that did not improve the model fit to the data. To find the best model we used the Akaike’s information criterion (AIC) [26]. Also, when necessary, levels were conflated within a given factor to construct the simplest model. GLMs with the R package *gmodels* [27] were performed within the statistical program R version 3.2.0 (R Development Core Team, 2015) [28].

## Results

### *Controrchis* spp.

Eggs of *Controrchis* spp. floated only using FS2, FS3, FS6 and FS7. The values of FS2, FS3 and FS6 were grouped into a single category, due to the low numbers of eggs detected in these solutions. Only in the FS7, eggs floated with both techniques at all dilution ratios. The likelihood of detection based on presence/absence vary significantly in relation to  apparatus (*χ* ^2^ = 5.5, *df* = 1, *P* = 0.019), preservation methods (*χ* ^2^ = 7.4, *df* = 1, *P* = 0.007) and FSs (*χ* ^2^ = 4.3, *df* = 1, *P* = 0.037). The samples preserved in 5% formalin and analyzed with FLOTAC with FS7 showed the best results with a probability of detection of 83.3% (*n* = 15/18) for *Controrchis* spp. infections.

**Fig. 2:**
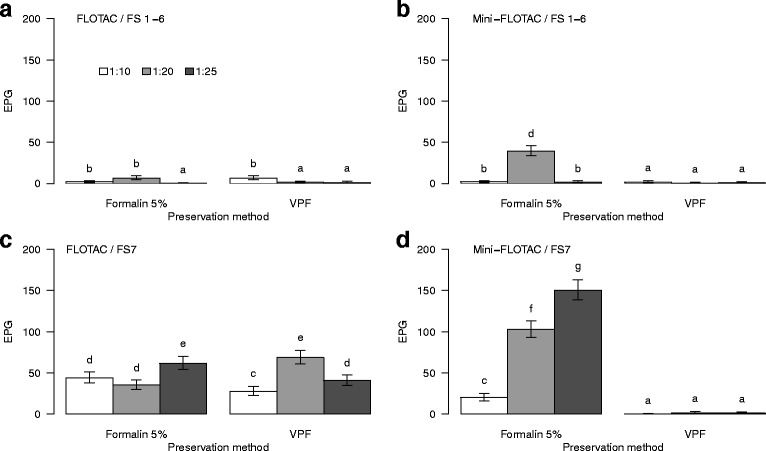
*Controrchis* spp. total egg counts. Comparison between calibration variables: methods, FSs, preservation methods and dilutions. **a** Total number of EPG using the FLOTAC technique with groups FS1-FS6. **b** Total number of EPG using the Mini-FLOTAC technique with groups FS1-FS6. **c** Total number of EPG using the FLOTAC technique with FS7. **d** Total number of EPG using the Mini-FLOTAC technique with FS7. Letters above the bars indicate the homogeneous groups based on the contrasts done. Differences for bars with the same letter were not statistically significant while those with different letters were statistically different. In all the bars the standard error is represented as a measure of dispersion around the mean

The evaluation based on counts of EPG in flotation differ in FSs (*χ* ^2^ = 445.4, *df* = 1, *P* < 0.001), preservation methods (*χ* ^2^ = 167.2, *df* = 1, *P* < 0.001) and dilution ratios (*χ* ^2^ = 85.2, *df* = 2, *P* < 0.001). All significant GLM results are shown in Additional file 5: Table S4. FS7 exhibited significantly larger counts than those obtained from the groups FS1-FS6 (Fig. 2). The highest values of EPG were obtained by 1:20 and 1:25 dilution, and 5% formalin also proved their efficiency in counts of EPG in flotation. The values of EPG in flotation was better using 5% formalin compared with VPF (*n* = 454 EPG, mean = 2.1 and *n* = 140 EPG, mean = 0.65, respectively).

Although there were no differences in relation to  apparatus (*χ* ^2^ = 1.1, *df* = 1, *P* = 0.296), Mini-FLOTAC was marginally better than FLOTAC based on counts of EPG in flotation (Fig. 2). The most appropriate combination of elements was (i) fecal samples preserved in 5% formalin and analyzed with FS7 at 1:20; and (ii) fecal samples preserved in 5% formalin and analyzed with FS7 at 1:25 dilution ratio. Using Mini-FLOTAC with the both previous combination an 83.3% of detection value is obtained for *Controrchis* spp. infections.

### *Trypanoxyuris* spp*.*

**Fig. 3:**
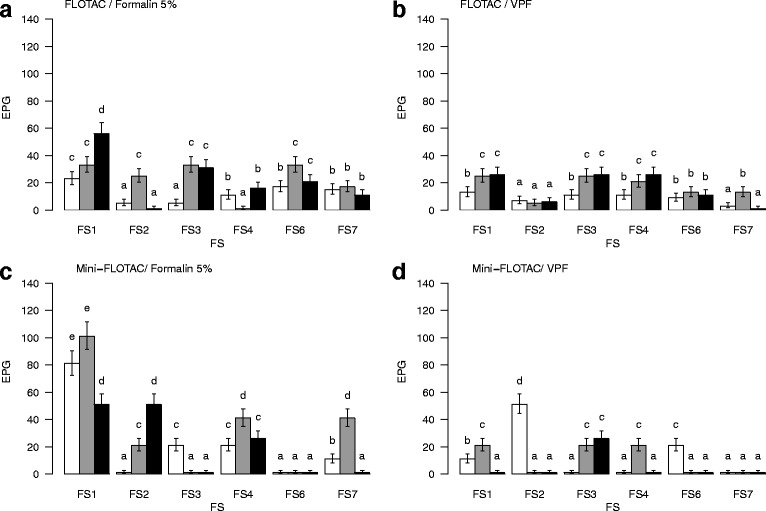
*Trypanoxyuris* spp. total egg counts. Comparison between calibration variables: methods, FSs, preservation methods and dilutions. **a** Total number of EPG using the FLOTAC technique with the six FS and 5% formalin as the preservation method. **b** Total number of EPG using the FLOTAC technique with the six FS and VPF as the preservation method. **c** Total number of EPG using the Mini-FLOTAC technique with the six FS and 5% formalin as the preservation method. **d** Total number of EPG using the Mini-FLOTAC technique with the six FS and VPF as the preservation method. Letters above the bars indicate the homogeneous groups based on the contrasts done. Differences for bars with the same letter were not statistically significant while those with different letters were statistically different. In all the bars the standard error is represented as a measure of dispersion around the mean

The eggs of *Trypanoxyuris* spp. floated with all the six FSs (Fig. 3) and there were differences in apparatus in detectability (*χ* ^2^ = 17.6, *df* = 1, *P* < 0.001). The FLOTAC method was, however, better than the Mini-FLOTAC method (50.4 and 13.8%, respectively) (Table 1).

**Table 1:** Generalized linear model output for *Trypanoxyuris* spp. detection (presence/absence)

	*df*	*χ* ^2^	*P*-value
FS	5	5.2	0.396
Apparatus	1	17.6	< 0.0001*
Dilution	2	4.1	0.131

*Significance at α = 0.05. Table reflects the final output of simplification of full model

Detection based on counts of EPG in flotation varied significantly among FSs (*χ* ^2^ = 286.8, *df* = 5, *P* < 0.001), preservation methods (*χ* ^2^ = 123.8, *df* = 1, *P* < 0.001), and dilution (*χ* ^2^ = 33.7, *df* = 2, *P* < 0.001). FS1 performed best with a significantly larger EPG count than FS2 (*z* = 3.1, *P* = 0.002), FS3 (*z* = 3.1, *P* = 0.002) and FS4 (*z* = 2.0, *P* = 0.044). Fecal samples preserved in 5% formalin detected more *Trypanoxyuris* spp. infections with a better EPG count in flotation than VPF. All significant GLM results are shown in (Additional file 6: Table S5).

Although there were no differences related to  apparatus (*χ* ^2^ = 2.0, *df* = 1, *P* = 0.159) based on counts of EPG in flotation, the combination Mini-FLOTAC method using FS1 at 1:10 dilution (80 EPG, mean = 13.3 EPG, SD = 5.2 EPG, CV = 38.7%) with samples preserved in 5% formalin performed the best EPG counts. This combination detected 100% of *Trypanoxyuris* spp. infections with less variation among samples compared to the same elements at 1:20 dilution (100 EPG, mean = 16.6 EPG, SD = 19.6 EPG, CV = 118.0%).

## Discussion

Although significant advances have been made in the parasitology and epidemiology of *Alouatta pigra* in the wild [5, 7–16], evaluation of the best methods to use were not available until now. We demonstrated that the likelihood of detecting a parasite and the egg count are dependent on the choice of preservation method, flotation technique, dilution ratio, and FS. In our study, many of the combinations of these elements were not adequate to detect the parasites and in a few specific combinations, the EPG counts were as much as 100 times higher, especially for *Controrchis* spp. These findings emphasize the need for standardization and calibration of the copro-microscopic techniques for an accurate detection and counting of parasites.

We do not know the real value of EPG of *Controrchis* spp. and *Trypanoxyuris* spp. infections although our study still provides useful guidelines. Infection levels reported in other studies of howler monkeys are generally low, the mean EPG of *Controrchis* spp. values reported using the sugar-flotation technique (SG = 1.2) by Kowalzik et al. [8] was 2.3 ± 1.9 (mean ± SD) and by Behie et al. [7] was 3.2 ± 1.4 eggs per gram of feces. Similarly, Behie et al. [7] reported an EPG value of 2.0 ± 1.4 for *Trypanoxyuris* spp. using sucrose solution (SG = 1.26). The general infection levels found in the present study using the best combination of elements for each parasite were 16.6 ± 8.1 EPG (with Mini-FLOTAC, FS7 at 1:20 dilution), and 25 ± 15.8 EPG (with Mini-FLOTAC, FS7 at 1:25 dilution) for *Controrchis* spp. and 13.3 ± 5.2 EPG for *Trypanoxyuris* spp. (with Mini-FLOTAC, FS1 at 1:10 dilution). For all combinations, fecal samples preserved in 5% formalin show significantly better results.

Here FLOTAC obtained the best results for detecting the presence of *Controrchis* spp. and *Trypanoxyuris* spp. infections. FLOTAC is a cylindrical device with two 5 ml flotation chambers, which allows up to 1 g of stool to be prepared for microscopic analysis [1]. However, species of the genus *Alouatta* are folivore-frugivore primates [19–21], and their feces have a high fiber content [19], with pollen, plant tissue, flowers, and other elements being abundant [19, 21, 22] and all this debris accumulates in the large chambers (5 ml) of FLOTAC hindering the quantitative analysis. As a result, we suggest the quantitative analysis of EPG using a Mini-FLOTAC as a better option. Mini-FLOTAC is considered in other studies, as the most sensitive method for detecting helminth infections compared with the formol-ether concentration and direct fecal smear methods for the diagnosis in humans [24, 29, 30]. It is also more sensitive than the McMaster (FEC technique) for the diagnosis of *Eimeria* in goats [31]. Barda et al. [24] reported a detection of 90% in helminth infections and 68% of protozoan infections. Also in the veterinary field Maurelli et al. [32], report a 100% of detection of the three most common intestinal nematodes in dogs (*Toxocara canis*, *Trichuris vulpis* and hookworm) with this method. In our study, with the appropriate combination of elements, Mini-FLOTAC performed good detection based on presence and EPG counts of both *Controrchis* spp. and *Trypanoxyuris* spp. avoiding the large amount of debris in the 5 ml of FLOTAC chambers.

Mini-FLOTAC is a simple technique (FEC technique) with two 1 ml flotation chambers, which are designed for the optimal examination of faecal sample suspensions (total volume = 2 ml) [24]. In our study, the probability of detection is higher (83.3% for detection of *Controrchis* spp. and 100% of *Trypanoxyuris* spp. infections) than in previous studies [24, 32].

The use of 5% formalin is recommended as this was associated with significantly higher egg detection and EPG counts for *Controrchis* spp. and *Trypanoxyuris* spp. infections compared with VPF. These findings are encouraging because in field conditions, high temperatures and humidity do not allow the preservation of fresh samples for later analysis and is effective to avoid degradation and loss of parasitic forms [33].

We suggest the use of zinc sulfate (SG = 1.35) for the detection of trematode eggs is the best option. For the detection of trematodes such as *Controrchis* spp., some authors use sedimentation techniques [12, 34], however, our results and those of previous studies [24, 35] showed that the flotation of this group of parasites can be achieved with FSs that have a high specific gravity. Also, Barda et al. [25] found that the FS7 is the most sensitive solution to detect *Ascaris lumbricoides* infections using the Mini-FLOTAC method, which is of interest because species of this parasite genus has also been found in howler monkeys [10, 36].

For nematodes such as *Trypanoxyuris* spp., FS1 (sucrose and formaldehyde; SG = 1.20) is recommended. These FSs have been used before on howler monkey fecal samples with different specific gravity [12, 13]. It is relevant to mention that, in this study, a popular solution used for copro-microscopic analysis of *Alouatta pigra*, FS2 [11, 18], provided a low detection of the parasites; *Trypanoxyuris* spp. were detected in 18.1% (*n* = 13/72) of samples and *Controrchis* spp. in 2.8% (*n* = 2/72) of infections. Similarly, our results showed that FS4, which is considered optimal for samples from wild primates [16, 17], was not suitable for the analysis of feces of howler monkeys, because it did not detect the presence of trematodes, and *Trypanoxyuris* spp. were detected only in 29.2% (*n* = 21/72) of samples.

If a qualitative or quantitative analysis of parasites of *Alouatta*is needed, we suggest Mini-FLOTAC with the different combination of elements shown (depending on the parasite group to be detected) is an appropriate method (Additional file 1). Our recommendations are made based on two species of parasites belonging to two parasitic groups (trematodes and nematodes), but the richness of parasites reported in *Alouatta* spp. is higher, thus similar studies evaluating different species are needed [24]. Finally, it was not possible to calibrate with fresh samples because FLOTAC technique needed specialized equipment (large volume or microtiter centrifuge) [23] that was not possible to use in the field. However, it is also important to compare different preservation methods against fresh samples [24, 37].

## Conclusions

This is the first in-depth calibration of fecal egg count flotation methods for analyzing samples from wild howler monkeys and should be applicable for comparisons of populations and species of howlers and other similar frugivore/folivores. The Mini-FLOTAC method is a promising technique for the qualificative and quantitative analysis of nematodes and trematodes in howler monkeys, and can be used in field without specialized equipment. For the trematode *Controrchis* spp. the highest EPG values were recorded with FS7 at 1:20 and 1:25 dilution; for the nematode *Trypanoxyuris* spp. the highest EPG values were recorded with FS1 at 1:10 dilution, for samples preserved in 5% formalin. These combinations achieved an 83.3% detection of *Controrchis* spp. and 100% of *Trypanoxyuris* spp. infections.

## Additional files

Additional file 1: Guide to the recommended quali-quantitative flotation method. (DOCX 14 kb)

Additional file 2: Table S1. Table showing in-depth calibration scheme. (DOCX 19 kb)

Additional file 3: Table S2. *Controrchis* spp. egg counts, stratified by flotation and preservation method, dilution and flotation solutions. (DOCX 19 kb)

Additional file 4: Table S3. *Trypanoxyuris* spp. egg counts, stratified by flotation and preservation method, dilution and flotation solutions. (DOCX 25 kb)

Additional file 5: Table S4. Generalized linear model output for *Controrchis* spp. egg counts. (DOCX 16 kb)

Additional file 6: Table S5. Generalized linear model output for *Trypanoxyuris* spp. egg counts. (DOCX 16 kb)

## Abbreviations

CV: Coefficient of variation
EPG: Eggs per gram of feces
EPG/LPG/OPG/CPG: Eggs/larvae/oocysts/cyst per gram of feces
FEC: Fecal egg count
FS1: Sucrose and formaldehyde/specific gravity SG = 1.2
FS2: Sodium chloride, SG = 1.2
FS3: Zinc sulfate, SG = 1.2
FS4: Sodium nitrate, SG = 1.2
FS6: Magnesium sulfate, SG = 1.28
FS7: Zinc sulfate, SG = 1.35
G: Gram
GLMs: Generalized linear models
SD: Standard deviation
VPF: Vacuum packing in a fridge (4 °C)
