# Supplementary material for: Flotation techniques (FLOTAC and mini-FLOTAC) for detecting gastrointestinal parasites in howler monkeys
Source: Parasit Vectors. 2017 Nov 23;10:586. doi: 10.1186/s13071-017-2532-7 (PMC5701314; doi:10.1186/s13071-017-2532-7)
Supplement: Supplementary file 1 — Guide to the recommended quali-quantitative flotation method. (DOCX 14 kb) [file 13071_2017_2532_MOESM1_ESM.docx]

**Additional file 1.** Guide to the recommended quali-quantitative flotation method

***Mini-FLOTAC Basic technique***

In the field weigh 5g of feces and add 15 ml of 5% formalin (proportion 1:4). Homogenize the sample. To analyze in a laboratory, homogenize the sample and filter for a 250 μm sieve. Take 2 ml (0.5g) of the fecal suspension for each of 2 tubes (4 ml/1g total). Centrifuge at 1500 rpm (170 RCF) for three minutes. Supernatant pour off and discard, leaving only a pellet in the tube. Add flotation solution (one tube until 5 ml of FS1 for the 1:10 dilution and second tube until 10 ml of FS7 for the 1:20 dilution). Homogenize the suspension and fill with 1 ml (0.1g) each chamber of Mini-FLOTAC using the filling holes, until a small meniscus is formed. Fill the two chambers with each FS, using two Mini-FLOTAC (one for FS1 and one for FS7). In order to avoid the formation of air bubbles, the chambers should be filled with the Mini-FLOTAC held at a slope. After 10 minutes, translate the reading disc and put the Mini-FLOTAC under the microscope, using the microscope adaptor. Examine and measure under a microscope with objectives 10 and 40 X for quantification of parasitic elements. This technique has the highest analytical sensitivity for Mini-FLOTAC procedure. The analytical sensitivity is 5 EPG for FS1 at 1:10 dilution and 10 EPG for FS7 at 1:20 dilution.

***Mini-FLOTAC dual technique***

In the field weigh 5g of feces and add 15 ml of 5% formalin (proportion 1:4). Homogenize the sample. To analyze in a laboratory, homogenize the sample and filter for a 250 μm sieve. Take 2 ml (0.5 g) of the fecal suspension for each of 2 tubes (4 ml/1g total). Centrifuge at 1500 rpm (170 RCF) for three minutes. Supernatant pour off and discard, leaving only a pellet in the tube. Add flotation solution (one tube until 5 ml of FS1 for the 1:10 dilution and second tube until 10 ml of FS7 for the 1:20 dilution). Homogenize the suspension and fill with 1 ml (0.1g) each chamber of Mini-FLOTAC (one chamber with FS1 and one with FS7), using the filling holes, until a small meniscus is formed. In order to avoid the formation of air bubbles, the chambers should be filled with the Mini-FLOTAC held at a slope. For this technique, one Mini-FLOTAC is used to analyze both solutions. After 10 minutes, translate the reading disc and put the Mini-FLOTAC under the microscope, using the microscope adaptor. Examine and measure under a microscope with objectives 10 and 40 X for quantification of parasitic elements. The analytical sensitivity in the dual technique is 10 EPG for FS1 at 1:10 dilution and 20 EPG for FS7 at 1:20 dilution. This technique is ideal when needed analyze a large number of samples, but if a considerable amount of samples is analyzed (~200 or less), the basic technique can be applied.
