# Supplementary material for: Flotation techniques (FLOTAC and mini-FLOTAC) for detecting gastrointestinal parasites in howler monkeys
Source: Parasit Vectors. 2017 Nov 23;10:586. doi: 10.1186/s13071-017-2532-7 (PMC5701314; doi:10.1186/s13071-017-2532-7)
Supplement: Supplementary file 2 — Table showing in-depth calibration scheme. (DOCX 19 kb) [file 13071_2017_2532_MOESM2_ESM.docx]

**Additional file 2: Table S1.**Table showing in-depth calibration scheme

|  |  |  |  | Composite sample | | | | |  |  |  |  |
| --- | --- | --- | --- | --- | --- | --- | --- | --- | --- | --- | --- | --- |
|  |  |  |  | (360 g) | | | | |  |  |  |  |
|  | Techniques / equipment | | | | | | | | | | | |
|  |  | FLOTAC | |  |  |  |  |  | Mini-FLOTAC | | |  |
|  | Preservation methods | | | | | | | | | | | |
|  | VPF | | | 5% formalin | | | VPF | | | 5% formalin | | |
|  | 90 g | | | 90 g | | | 90 g | | | 90 g | | |
|  | Dilutions | | | | | | | | | | | |
| Solution | 1,10 | 1,20 | 1,25 | 1,10 | 1,20 | 1,25 | 1,10 | 1,20 | 1,25 | 1,10 | 1,20 | 1,25 |
| FS1 | 6 tubes (6 ml) | 6 | 6 | 6 | 6 | 6 | 6 | 6 | 6 | 6 | 6 | 6 |
| FS2 | 6 | 6 | 6 | 6 | 6 | 6 | 6 | 6 | 6 | 6 | 6 | 6 |
| FS3 | 6 | 6 | 6 | 6 | 6 | 6 | 6 | 6 | 6 | 6 | 6 | 6 |
| FS4 | 6 | 6 | 6 | 6 | 6 | 6 | 6 | 6 | 6 | 6 | 6 | 6 |
| FS6 | 6 | 6 | 6 | 6 | 6 | 6 | 6 | 6 | 6 | 6 | 6 | 6 |
| FS7 | 6 | 6 | 6 | 6 | 6 | 6 | 6 | 6 | 6 | 6 | 6 | 6 |
| Total | 36 tubes | 36 | 36 | 36 | 36 | 36 | 36 | 36 | 36 | 36 | 36 | 36 |

*Flotation solutions: FS1* sucrose and formaldehyde SG=1.20; *FS2* sodium chloride SG=1.20; *FS3* zinc sulfate SG=1.20; *FS4* sodium nitrate SG=1.20; *FS6* magnesium sulfate SG=1.28; *FS7* zinc sulfate SG=1.35; *VPF* vacuum packing in the fridge (4°C)
