# Supplementary material for: Flotation techniques (FLOTAC and mini-FLOTAC) for detecting gastrointestinal parasites in howler monkeys
Source: Parasit Vectors. 2017 Nov 23;10:586. doi: 10.1186/s13071-017-2532-7 (PMC5701314; doi:10.1186/s13071-017-2532-7)
Supplement: Supplementary file 3 — Controrchis spp. egg counts, stratified by flotation and preservation method, dilution and flotation solutions. (DOCX 19 kb) [file 13071_2017_2532_MOESM3_ESM.docx]

**Additional file 3: Table S2.** *Controrchis* spp. egg counts, stratified by flotation and preservation method, dilution and flotation solutions

|  | | Flotation solution | | | | | | |
| --- | --- | --- | --- | --- | --- | --- | --- | --- |
|  |  | FS2 | FS3 | | FS6 | FS7 | | |
|  | Dilution | 1:20 | 1:10 | 1:20 | 1:10 | 1:10 | 1:20 | 1:25 |
| FLOTAC:PF | EPG |  | 6 |  |  | 26 | 68 | 40 |
|  | Mean |  | 1 |  |  | 4.333 | 11.333 | 6.666 |
|  | *SD* |  | 1.673 |  |  | 1.966 | 5.877 | 7.527 |
|  | CV |  | 167.332 |  |  | 45.378 | 51.951 | 112.915 |
| FLOTAC: 5% Formalin | EPG | 4 |  |  | 2 | 42 | 36 | 60 |
|  | Mean | 0.666 |  |  | 0.333 | 7 | 6 | 10 |
|  | *SD* | 1.632 |  |  | 0.816 | 2.756 | 8.294 | 5.477 |
|  | CV | 244.948 |  |  | 244.948 | 39.382 | 138.242 | 54.772 |
| Mini-FLOTAC: Formalin 5% | EPG | 20 |  | 20 |  | 20 | 100 | 150 |
|  | Mean | 3.333 |  | 3.333 |  | 3.333 | 16.666 | 25 |
|  | *SD* | 8.164 |  | 8.164 |  | 5.163 | 8.164 | 15.811 |
|  | CV | 244.948 |  | 244.948 |  | 154.919 | 48.989 | 63.245 |

*EPG* eggs per gram of feces, *Mean* eggs per gram of feces / six repetitions , *SD*  standard Deviation, *CV* coefficient of variation (%), *FS2* sodium chloride SG=1.20; *FS3* zinc sulfate SG=1.20; *FS6* magnesium sulfate SG=1.28; *FS7* zinc sulfate SG=1.35; *VPF* vacuum packing in the fridge (4°C).
