# Supplementary material for: Flotation techniques (FLOTAC and mini-FLOTAC) for detecting gastrointestinal parasites in howler monkeys
Source: Parasit Vectors. 2017 Nov 23;10:586. doi: 10.1186/s13071-017-2532-7 (PMC5701314; doi:10.1186/s13071-017-2532-7)
Supplement: Supplementary file 4 — Trypanoxyuris spp. egg counts, stratified by flotation and preservation method, dilution and flotation solutions. (DOCX 25 kb) [file 13071_2017_2532_MOESM4_ESM.docx]

**Additional file 4: Table S3.** *Trypanoxyuris* spp. egg counts, stratified by flotation and preservation method, dilution and flotation solutions

|  | | Flotation solution | | | | | | | | |
| --- | --- | --- | --- | --- | --- | --- | --- | --- | --- | --- |
|  |  | FS1 | | | FS2 | | | FS3 | | |
|  | Dilution | 1:10 | 1:20 | 1:25 | 1:10 | 1:20 | 1:25 | 1:10 | 1:20 | 1:25 |
| FLOTAC: VPF | EPG | 12 | 24 | 25 | 6 | 4 | 5 | 10 | 24 | 25 |
|  | Mean | 2 | 4 | 4.166 | 1 | 0.666 | 0.833 | 1.666 | 4 | 4.166 |
|  | *SD* | 1.788 | 2.529 | 3.763 | 1.673 | 1.632 | 2.041 | 0.816 | 5.059 | 3.763 |
|  | CV | 89.442 | 63.245 | 90.332 | 167.332 | 244.948 | 244.948 | 48.989 | 126.491 | 90.332 |
| FLOTAC: Formalin 5% | EPG | 22 | 32 | 55 | 4 | 24 |  | 4 | 32 | 30 |
|  | Mean | 3.666 | 5.333 | 9.166 | 0.666 | 4 |  | 0.666 | 5.333 | 5 |
|  | *SD* | 0.816 | 3.265 | 4.915 | 1.632 | 6.196 |  | 1.032 | 5.465 | 3.162 |
|  | CV | 22.268 | 61.237 | 53.628 | 244.948 | 154.919 |  | 154.919 | 102.469 | 63.245 |
| Mini-FLOTAC: VPF | EPG | 10 | 20 |  | 50 |  |  |  | 20 | 25 |
|  | Mean | 1.666 | 3.333 |  | 8.333 |  |  |  | 3.333 | 4.166 |
|  | *SD* | 4.082 | 8.164 |  | 7.527 |  |  |  | 8.164 | 10.206 |
|  | CV | 244.948 | 244.948 |  | 90.332 |  |  |  | 244.948 | 244.948 |
| Mini-FLOTAC: Formalin 5% | EPG | 80 | 100 | 50 |  | 20 | 50 | 20 |  |  |
|  | Mean | 13.333 | 16.666 | 8.333 |  | 3.333 | 8.333 | 3.333 |  |  |
|  | *SD* | 5.163 | 19.663 | 20.412 |  | 8.164 | 20.412 | 5.163 |  |  |
|  | CV | 38.729 | 117.983 | 244.948 |  | 244.948 | 244.948 | 154.919 |  |  |

|  | |  | | | Flotation solution | | |  | | |
| --- | --- | --- | --- | --- | --- | --- | --- | --- | --- | --- |
|  |  | FS4 | | | FS6 | | | FS7 | | |
|  | Dilution | 1:10 | 1:20 | 1:25 | 1:10 | 1:20 | 1:25 | 1:10 | 1:20 | 1:25 |
| FLOTAC: VPF | EPG | 10 | 20 | 25 | 8 | 12 | 10 | 2 | 12 |  |
|  | Mean | 1.666 | 3.333 | 4.166 | 1.333 | 2 | 1.666 | 0.333 | 2 |  |
|  | *SD* | 2.338 | 5.316 | 4.915 | 1.032 | 2.19 | 2.581 | 0.816 | 2.19 |  |
|  | CV | 140.285 | 159.499 | 117.983 | 77.459 | 109.544 | 154.919 | 244.948 | 109.544 |  |
| FLOTAC: Formalin 5% | EPG | 10 |  | 15 | 16 | 32 | 20 | 14 | 16 | 10 |
|  | Mean | 1.666 |  | 2.5 | 2.666 | 5.333 | 3.333 | 2.333 | 2.666 | 1.666 |
|  | *SD* | 1.505 |  | 2.738 | 1.032 | 6.022 | 4.082 | 1.966 | 4.131 | 2.581 |
|  | CV | 90.332 |  | 109.544 | 38.729 | 112.915 | 122.474 | 84.273 | 154.919 | 154.919 |
| Mini-FLOTAC: VPF | EPG |  | 20 |  | 20 |  |  |  |  |  |
|  | Mean |  | 3.333 |  | 3.333 |  |  |  |  |  |
|  | *SD* |  | 8.164 |  | 5.163 |  |  |  |  |  |
|  | CV |  | 244.948 |  | 154.919 |  |  |  |  |  |
| Mini-FLOTAC: Formalin 5% | EPG | 20 | 40 | 25 |  |  |  | 10 | 40 |  |
|  | Mean | 3.333 | 6.666 | 4.166 |  |  |  | 1.666 | 6.666 |  |
|  | *SD* | 5.163 | 10.327 | 10.206 |  |  |  | 4.082 | 10.327 |  |
|  | CV | 154.919 | 154.919 | 244.948 |  |  |  | 244.948 | 154.919 |  |

*EPG* eggs per gram of feces, *Mean* eggs per gram of feces / six repetitions, *SD* standard Deviation, *CV* coefficient of variation (%); *VPF* vacuum packing in the fridge (4°C); *FS1* sucrose and formaldehyde SG=1.20 ; *FS2* sodium chloride SG=1.20 ; *FS3* zinc sulfate SG=1.20; *FS4* sodium nitrate SG=1.20 ; *FS6* magnesium sulfate SG=1.28 ; *FS7* zinc sulfate SG=1.35.
