# Supplementary material for: Flotation techniques (FLOTAC and mini-FLOTAC) for detecting gastrointestinal parasites in howler monkeys
Source: Parasit Vectors. 2017 Nov 23;10:586. doi: 10.1186/s13071-017-2532-7 (PMC5701314; doi:10.1186/s13071-017-2532-7)
Supplement: Supplementary file 5 — Generalized linear model output for Controrchis spp. egg counts. (DOCX 16 kb) [file 13071_2017_2532_MOESM5_ESM.docx]

**Additional file 4: Table S4.** Generalized linear model output for *Controrchis* spp. egg counts.

|  | *df* | *X^2^* | p-value |
| --- | --- | --- | --- |
| FS | 1 | 445.4 | **<0.0001** |
| Apparatus | 1 | 1.09 | 0.296 |
| Preservation methods | 1 | 167.2 | **<0.0001** |
| Dilution | 2 | 85.2 | **<0.0001** |
| FS:Apparatus | 1 | 11.6 | **0.0007** |
| FS: Preservation methods | 1 | 1.39 | 0.239 |
| FS:Dilution | 2 | 48.7 | **<0.0001** |
| Apparatus:Preservation methods | 1 | 219.2 | **<0.0001** |
| Apparatus:Dilution | 2 | 48.8 | **<0.0001** |
| Preservation methods:Dilution | 2 | 10.1 | **0.0006** |
| FS: Apparatus: Preservation methods | 1 | 3.02 | 0.082 |
| Apparatus: Preservation methods: Dilution | 2 | 1.12 | 0.570 |
| FS:Preservation methods:Dilution | 2 | 26.3 | **<0.0001** |

Bold indicates significance at α = 0.05. Table reflects the final output of simplification of full model.
