# Supplementary material for: Flotation techniques (FLOTAC and mini-FLOTAC) for detecting gastrointestinal parasites in howler monkeys
Source: Parasit Vectors. 2017 Nov 23;10:586. doi: 10.1186/s13071-017-2532-7 (PMC5701314; doi:10.1186/s13071-017-2532-7)
Supplement: Supplementary file 6 — Generalized linear model output for Trypanoxyuris spp. egg counts. (DOCX 16 kb) [file 13071_2017_2532_MOESM6_ESM.docx]

**Additional file 5: Table S5.** Generalized linear model output for *Trypanoxyuris* spp. egg counts

|  | *df* | *X^2^* | p-value |
| --- | --- | --- | --- |
| FS | 5 | 286.8 | **<0.0001** |
| Apparatus | 1 | 2.0 | 0.159 |
| Preservation methods | 1 | 123.8 | **<0.0001** |
| Dilution | 2 | 33.7 | **<0.0001** |
| FS:Apparatus | 5 | 123.1 | **<0.0001** |
| FS:Preservation methods | 5 | 94.5 | **<0.0001** |
| FS:Dilution | 10 | 61.4 | **<0.0001** |
| Apparatus:Preservation methods | 1 | 16.9 | **<0.0001** |
| Apparatus:Dilution | 2 | 39.6 | **<0.0001** |
| Preservation methods:Dilution | 2 | 15.8 | **0.0003** |
| FS:Apparatus:Preservation methods | 5 | 111.6 | **<0.0001** |
| FS:Apparatus:Dilution | 10 | 104.0 | **<0.0001** |
| FS:Preservation methods:Dilution | 10 | 162.3 | **<0.0001** |
| Apparatus:Preservation methods:Dilution | 2 | 1.9 | 0.396 |
| FS:Apparatus:Preservation methods:Dilution | 10 | 106.8 | **<0.0001** |

Bold indicates significance at α = 0.05. Table reflects the final output of simplification of full model.
